# Two Drug–Drug Co-Amorphous Systems of Curcumin and Berberine Hydrochloride/Palmatine Hydrochloride with Improved Physicochemical Properties and Multifunctional Activities

**DOI:** 10.3390/pharmaceutics18010009

**Published:** 2025-12-20

**Authors:** Yanjie Zhang, Quanhu Guo, Ling Liang, Mei Zhang, Rongjian Sa, Benyong Lou

**Affiliations:** College of Materials and Chemical Engineering, Minjiang University, Fuzhou 350108, China; 2268271457@qq.com (Q.G.); liangling@mju.edu.cn (L.L.); zhangmei11@163.com (M.Z.); rjsa@mju.edu.cn (R.S.)

**Keywords:** curcumin, co-amorphous system, berberine hydrochloride, palmatine hydrochloride, physical stability, solubility, antioxidant activity, anticancer activity

## Abstract

**Background/Objectives**: The poor aqueous solubility of curcumin (CUR) limits its pharmaceutical application. Although amorphization can enhance its solubility, the amorphous form often exhibits insufficient physical stability. Co-amorphization, particularly drug–drug co-amorphous (CAM) formation, offers a promising approach to improve solubility, stability, and therapeutic efficacy. This study aimed to prepare and evaluate two CUR-based CAM systems using isoquinoline alkaloids berberine hydrochloride (BER) and palmatine hydrochloride (PAL) as co-formers to achieve simultaneous stabilization and synergistic bioactivity. **Methods**: CUR-BER and CUR-PAL CAM systems were prepared via rotary evaporation under vacuum at a 1:1 molar ratio. The solid-state properties were characterized by powder X-ray diffraction (PXRD), differential scanning calorimetry (DSC), scanning electron microscope (SEM), and ^13^C solid-state nuclear magnetic resonance spectroscopy (ssNMR). Dissolution, solubility, and stability studies were conducted, while antioxidant and anticancer activities were assessed by DPPH/ABTS^+^ radical-scavenging and MTT assays using HT-29 colorectal cancer cells. **Results**: PXRD and DSC confirmed the formation of single-phase amorphous systems with higher glass transition temperatures, indicating strong intermolecular interactions between CUR and BER/PAL. ^13^C ssNMR spectroscopy evidenced hydrogen-bond formation between the enolic hydroxyl moiety of CUR and the methoxy oxygen atoms in BER or PAL molecules. Both CAM systems significantly enhanced the solubility and dissolution rate of CUR, with CUR-PAL CAM showing up to a 15.1-fold solubility improvement. The CAM systems also displayed superior thermal stability, photolytic stability, and improved short-term humidity resistance, together with enhanced antioxidant and anticancer activities compared with pure amorphous CUR. **Conclusions**: Co-amorphization of CUR with isoquinoline alkaloids effectively improved solubility, stability, antioxidant and anticancer activities, representing a promising strategy for the rational design of multifunctional amorphous CUR-based drug formulations.

## 1. Introduction

The modification of solid dosage forms has attracted increasing attention in the pharmaceutical industry as a strategy to enhance the solubility of active pharmaceutical ingredients (APIs) [1]. Various solid-state forms, including salts, solvates, low-melting mixtures, co-crystals, and amorphous forms, have been explored [2,3]. Due to their higher solubility, amorphous forms have emerged as an effective approach to address the poor solubility of many APIs [4]. However, the intrinsic physical instability of amorphous forms during manufacturing and storage often leads to recrystallization, which significantly restricts their commercial application [5]. Notably, co-amorphous (CAM) systems can overcome these limitations by improving the physicochemical properties and stability of APIs [6]. A co-amorphous system is defined as a single-phase amorphous material characterized by a single glass transition temperature (T_g_), formed by combining APIs with small molecules such as other drugs or excipients. The physical stability of these systems is primarily enhanced by intermolecular interactions, particularly hydrogen bonding, between the API and the co-former [7,8]. Of special interest are drug–drug co-amorphous systems, which can simultaneously improve the efficacy of one API while reducing the toxicity or side effects of another [9,10].

Curcumin (CUR), a phytochemical with a favorable safety profile and minimal side effects, has been extensively studied. It possesses a broad spectrum of biological activities, including antioxidant, anticancer, antibacterial, antiviral, and neuroprotective effects [11,12,13,14]. Nevertheless, its clinical application has been limited by its extremely low solubility and poor bioavailability [13,14]. To achieve adequate oral absorption, high doses of CUR are often required, which may cause gastrointestinal side effects such as diarrhea, headache, rash, and yellow stool [15]. Several co-amorphous forms of CUR have been reported, such as CUR-piperine [16,17], CUR-myricetin [18], CUR-L-arginine [19], and CUR-acetyl salicylic acid [20], all of which demonstrated superior dissolution rates compared with pure CUR. Co-amorphization can also improve the stability of CUR and the co-former [16], achieve the simultaneous release of both components [16], enhance the bioavailability of CUR [17,18,19], and produce synergistic pharmacological effects such as anti-inflammatory [19,20] and anticancer [21] activities. The phenolic hydroxyl, enolic C-OH, and methoxyl groups of curcumin are generally regarded as key functional sites that can act as hydrogen bond donors or acceptors during interactions with coformers [16,19,22].

**Figure 1:**
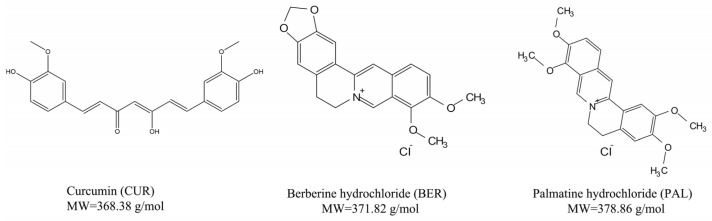
Molecular structures and molecular weights of CUR, BER, and PAL.

Berberine hydrochloride (BER), an isoquinoline alkaloid derived from *Rhizoma coptidis*, has long been used in traditional Chinese medicine. It exhibits diverse pharmacological activities, including antibacterial, hypolipidemic, anti-inflammatory, anticancer, and anti-obesity effects [23,24]. Palmatine hydrochloride (PAL), a structurally related natural isoquinoline alkaloid, also possesses antibacterial, anti-inflammatory, and anticancer properties [25,26]. The structural similarity of BER and PAL lies in their rigid skeletons and multiple oxygen atoms, which serve as strong hydrogen bond acceptors. These oxygen atoms can engage in hydrogen bonding with functional groups such as carboxyl, sulfonyl, and hydroxyl groups [27,28,29]. Therefore, the use of oxygen-containing alkaloids such as BER and PAL as co-formers with CUR provides a promising strategy for the design of CUR-based drug–drug co-amorphous systems. The molecular structures and molecular weights (MW) of CUR, BER, and PAL are shown in Figure 1.

This study aims to design and develop two curcumin-based drug–drug co-amorphous systems using berberine hydrochloride (BER) and palmatine hydrochloride (PAL) as co-formers, with the objectives of improving curcumin’s solubility and physical stability while achieving potential synergistic bioactivity. To accomplish this, the proposed work includes: (i) preparation of CUR-BER and CUR-PAL co-amorphous systems via solvent evaporation; (ii) comprehensive solid-state characterization using PXRD, DSC, SEM and ssNMR to confirm amorphization and elucidate intermolecular interactions; (iii) evaluation of their dissolution behavior and equilibrium solubility under physiologically relevant conditions; (iv) assessment of physical stability under thermal, humidity, and light stress; and (v) preliminary investigation of antioxidant and anticancer activities to explore synergistic potential. This structured workflow is designed to establish the feasibility and multifunctional advantages of these CUR-based co-amorphous systems.

## 2. Materials and Methods

### 2.1. Materials

Curcumin (CUR, purity ≥ 98%), berberine hydrochloride dihydrate (BER·2H_2_O, purity ≥ 97%), and palmatine hydrochloride trihydrate (PAL·3H_2_O, purity ≥ 97%) were purchased from Dalian Meilun Biotechnology Co., Ltd. (Dalian, China). Methanol (purity ≥ 99.7%) was obtained from Sinopharm Chemical Reagent Co., Ltd. (Shanghai, China). Tween 80, DPPH (purity ≥ 97%) and ABTS (purity ≥ 98%) were purchased from Shanghai Macklin Biochemical Co., Ltd. (Shanghai, China). Cell culture media (Gibco RPMI-1640 medium supplemented with 10% FBS) were purchased from Thermo Fisher Scientific Inc. (Waltham, MA, USA). All chemicals were used without further purification.

### 2.2. Preparation of Amorphous and Co-Amorphous Samples

Amorphous CUR was prepared by solvent evaporation under reduced pressure using a rotary evaporator. Specifically, 100 mg of CUR was dissolved in 20 mL of methanol, and the solvent was subsequently removed using a rotary vacuum evaporator at 55 °C. Co-amorphous samples were prepared in a similar manner. CUR and BER (or PAL) were mixed at a 1:1 molar ratio (0.3 mmol each) and dissolved in 30 mL of methanol. The solvent was removed using a rotary vacuum evaporator at 55 °C. All obtained samples were further dried under vacuum at 60 °C for 12 h to remove residual solvents and then stored in a vacuum desiccator at room temperature until further use. The two co-amorphous samples were designated as CUR-BER CAM and CUR-PAL CAM, respectively.

### 2.3. Powder X-Ray Diffraction (PXRD)

PXRD patterns of all solid samples were recorded on a SmartLab X-ray diffractometer (Rigaku, Tokyo, Japan) using Cu Kα radiation at 40 kV and 40 mA. Data were collected in reflection mode over a 2θ range of 3–50° at a scanning speed of 20°/min with a step size of 0.02°.

### 2.4. Differential Scanning Calorimetry (DSC)

DSC measurements were performed on a DSC214 instrument (Netzsch, Waldkraiburg, Germany). Approximately 5 mg of each sample was sealed in an aluminum pan with a pinhole lid and heated from 30 to 300 °C at a rate of 10 °C/min under a nitrogen purge (20 mL/min).

### 2.5. Scanning Electron Microscopy (SEM)

The morphology of the samples was examined using an SU-8000 field-emission scanning electron microscope (Hitachi, Tokyo, Japan). A cold field emission source was employed, with an accelerating voltage of 5.0 kV and an accelerating current of 5 μA, and the images were captured at magnifications ranging from 500 to 5000.

### 2.6. ^13^C Solid-State Nuclear Magnetic Resonance Spectroscopy (ssNMR)

Solid-state ^13^C NMR spectra were obtained on a Bruker AVANCE III spectrometer (Bruker, Mannheim, Germany) operating at 400 MHz. Cross-polarization (CP) techniques, high-power TPPM decoupling, and magic angle spinning (MAS) were employed to enhance sensitivity. The sample was packed into a 4 mm rotor and spun at 10 kHz. The KBr method was used to calibrate the magic angle. The ^1^H-^13^C cross-polarization magic-angle spinning (CP/MAS) experiment (ν_rot_ = 10 kHz), performed with high-power ^1^H decoupling, was conducted under the condition ω_1H_/2π = γ_H_H_1H_/2π = 60 kHz. The recycle delay was set at 6.5 s. Data were acquired with an H/X dual resonance solid-state probe in TOSS mode at a resonance frequency of 100.625 MHz. Each spectrum was obtained from 480 scans, providing adequate signal-to-noise ratios. All experiments were conducted at room temperature.

### 2.7. In Vitro Dissolution Test

The release behavior was evaluated using an RC806ADK dissolution tester (Tianjin Tianda Tianfa, Tianjin, China) in a water bath maintained at 37 °C, with a stirring speed of 100 rpm (Chinese Pharmacopoeia paddle method) [30]. To ensure uniformity, samples were sieved through a 100-mesh screen (mesh size 0.15 mm). An amount equivalent to 50 mg CUR was dispersed in 250 mL of deionized water containing 0.5% Tween-80. At predetermined intervals, 2 mL of suspension was withdrawn, filtered through a 0.45 μm nylon membrane, and analyzed by HPLC. Each test was performed in triplicate.

### 2.8. Equilibrium Solubility

Equilibrium solubility was determined using the shake-flask method. The dissolution media included deionized water (with or without 0.5% Tween-80), 0.1 M hydrochloric acid (pH 1.2, with or without 0.5% Tween-80), 0.05 M phosphate-buffered saline (PBS, pH 6.8, with or without 0.5% Tween-80), and 0.05 M sodium acetate acetic acid buffer (pH 4.0, with or without 0.5% Tween-80). These media were selected to simulate different physiological environments and to assess the pH-dependent dissolution characteristics of the formulations. A total of 100 mg of each sample was added to 2 mL of dissolution medium. Suspensions were sonicated for 10 min and then shaken at 37 °C for 48 h to reach equilibrium. The samples were centrifuged, and the supernatants were filtered through 0.45 μm nylon membranes. After appropriate dilution, CUR, BER, and PAL concentrations were measured by HPLC. All tests were performed in triplicate.

### 2.9. High-Performance Liquid Chromatography (HPLC)

Concentrations of CUR, BER, and PAL were quantified using a Shimadzu LC-20AD HPLC system (Shimadzu, Kyoto, Japan) equipped with a Shim-pack GIST C18 column (4.6 × 150 mm, 5 μm). The mobile phase consisted of methanol (A) and 0.2% aqueous phosphoric acid solution (B) in a 70:30 ratio, delivered at 1.0 mL/min. The column oven was maintained at 30 °C, and the injection volume was 5 μL. Detection wavelengths were set at 430 nm for CUR and 415 nm for BER or PAL.

### 2.10. Physical Stability

To evaluate physical stability, amorphous CUR, CUR-BER CAM, and CUR-PAL CAM samples were stored under stress conditions. Samples were placed in a SHH-100GD-2 photostability chamber (Chongqing Yongsheng, Chongqing, China) at 4500 Lx light intensity (for 90 days), or in a LHH-150SD stability test chamber (Shanghai Yiheng, Shanghai, China) at 60 °C (for 90 days) or 40 °C/75% RH (for 24 h). PXRD was performed at predetermined intervals to monitor recrystallization.

### 2.11. DPPH Radical-Scavenging Activity

The DPPH assay was performed following a published method [31]. A 6 × 10^−5^ M DPPH solution was prepared in ethanol, and subsequently, 100 μL of this solution was mixed with 100 μL of sample solution at varying concentrations in a 96-well microtiter plate. After incubation in the dark at room temperature for 30 min, the absorbance was measured at 517 nm using a Spark microplate reader (Tecan, Männedorf, Switzerland). Radical scavenging activity was calculated as inhibition rate (%) = [(A_B_ − A_S_)/A_B_] × 100%, where A_B_ and A_S_ denote the absorbance values of the blank and sample, respectively. All measurements were conducted in triplicate, and results are expressed as mean ± SD.

### 2.12. ABTS^+^ Radical-Scavenging Activity

ABTS^+^ radical scavenging was assessed using a literature method [31]. ABTS^+^ solution was generated by reacting 7 mM ABTS with 2.54 mM potassium persulfate, incubated in the dark for 16 h at room temperature. The working solution was diluted with ethanol to an absorbance of 0.70 ± 0.02 at 734 nm. Test samples (100 μL) were mixed with ABTS^+^ solution (100 μL) in a 96-well microtiter plate. After 5 min, absorbance was measured at 734 nm, and radical scavenging activity was calculated using the same formula as in the DPPH assay. All experiments were performed in triplicate.

### 2.13. Anticancer Activity

The anticancer activity was evaluated using the MTT assay in HT-29 cells (sourced from the National Collection of Authenticated Cell Cultures of China, accession number HTB-38). Cells were seeded into 96-well plates at 4.0 × 10^3^ cells/well and incubated overnight. The cells were then treated with varying concentrations of samples for 24, 48, or 72 h. After treatment, 20 μL of MTT solution (5 mg/mL, solution in PBS) was added to each well, and the plates were incubated at 37 °C for 4 h. After removing the supernatant, 200 μL of DMSO was added to each well, and the plates were shaken gently for 10 min. The optical density (OD) of each well was measured by a microplate reader at 570 nm. The inhibition rate (IR) was calculated as inhibition rate (%) = (1 − OD_treatment_/OD_control_) × 100%. All experiments were performed in triplicate.

### 2.14. Statistical Analysis

The results of radical-scavenging and antitumor assays are presented as the mean ± standard deviation (SD). Statistical analysis was performed using Student’s *t*-test or one-way ANOVA with Origin 2018 software (version 9.5, OriginLab Corporation, Northampton, MA, USA). Differences were considered statistically significant at *p* < 0.05 (* *p* < 0.05, ** *p* < 0.01, *** *p* < 0.001).

## 3. Results and Discussion

### 3.1. Solid-State Characterization by PXRD, DSC, and SEM

**Figure 2:**
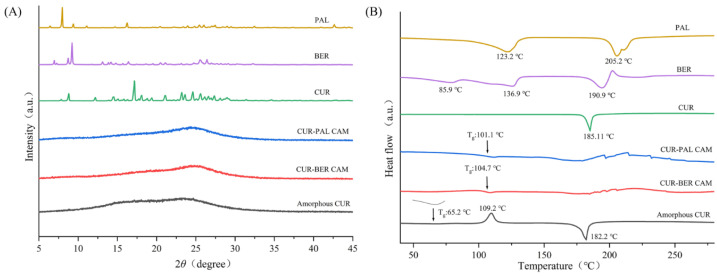
PXRD patterns (**A**) and DSC curves (**B**) of CUR, BER, PAL, amorphous CUR, CUR-BER CAM, and CUR-PAL CAM.

As shown in Figure 2A, the PXRD pattern of raw materials (CUR, BER, and PAL) displayed sharp diffraction peaks characteristic of a highly ordered crystalline lattice. In contrast, CUR obtained by rotary evaporation (amorphous CUR) exhibited broad and diffuse halos without distinct reflections, indicating an amorphous state. Similarly, both CUR-BER CAM and CUR-PAL CAM (both at a 1:1 molar ratio) showed no crystalline diffraction peaks, confirming the absence of long-range order and suggesting successful amorphization of the samples. The PXRD results of the samples prepared at other CUR-to-BER/PAL molar ratios (2:1 and 1:2) showed that all formulations exhibited amorphous characteristics, except for the CUR-BER system at the 1:2 molar ratio (Figure S1).

DSC analysis was further employed to confirm the amorphous nature of the samples. As shown in Figure 2B, the DSC thermogram of amorphous CUR differed markedly from that of crystalline CUR. Crystalline CUR exhibited a single endothermic melting peak at 185.1 °C. In contrast, amorphous CUR displayed a glass transition temperature (T_g_) at 65.2 °C. This was followed by an exothermic recrystallization peak at 109.2 °C and an endothermic melting peak at 182.2 °C, reflecting the inherent thermodynamic tendency of amorphous CUR to recrystallize. By contrast, the DSC thermograms of CUR-BER CAM and CUR-PAL CAM did not show the characteristic recrystallization peak observed in amorphous CUR. Instead, both samples exhibited only a broad endothermic event in the range of 150–200 °C, further supporting their amorphous character and indicating that recrystallization was effectively suppressed.

More importantly, CUR-BER CAM and CUR-PAL CAM each exhibited a single T_g_, measured at 104.7 °C and 101.1 °C, respectively. The presence of a single T_g_ strongly indicates homogeneous single-phase amorphous systems, i.e., co-amorphous forms [32]. The markedly higher T_g_ values compared with amorphous CUR suggest enhanced molecular-level stabilization and improved thermal stability. This effect is most likely attributable to intermolecular interactions, such as probable hydrogen bonding between CUR and BER/PAL, which reduce molecular mobility and hinder rearrangement into a crystalline lattice. Such stabilization is consistent with the superior thermal stability of CUR-BER CAM and CUR-PAL CAM observed in subsequent physical stability studies.

**Figure 3:**
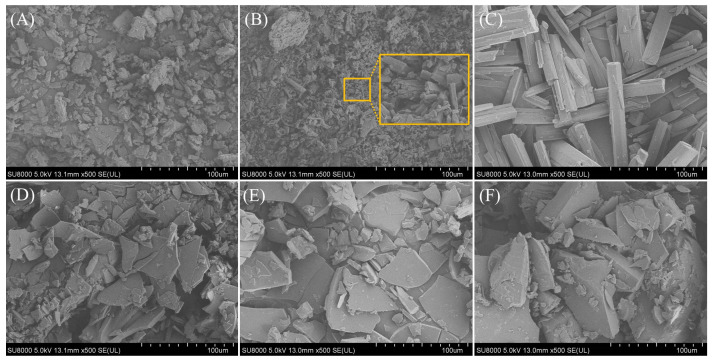
SEM images of CUR (**A**), BER (**B**), PAL (**C**), amorphous CUR (**D**), CUR-BER CAM (**E**), and CUR-PAL CAM (**F**).

As presented in Figure 3, the SEM results clearly distinguish the morphological characteristics of the crystalline raw materials from those of the amorphous and co-amorphous systems. The crystalline CUR exhibits irregular particle morphology, while BER displays relatively regular block-like morphology, as illustrated by the enlarged image of the yellow-highlighted region in Figure 3B. PAL shows well-defined rod-like morphology consistent with its intrinsic crystalline structure. In contrast, amorphous CUR, CUR-BER CAM, and CUR-PAL CAM all present featureless, collapsed, and irregular morphologies, which are representative of amorphous/co-amorphous solids lacking long-range molecular order. These observations are consistent with the PXRD and DSC results.

Together, the PXRD, DSC, and SEM data provide compelling evidence that CUR-BER CAM and CUR-PAL CAM form stable co-amorphous systems.

### 3.2. Intermolecular Interaction Analysis by ^13^C ssNMR

**Figure 4:**
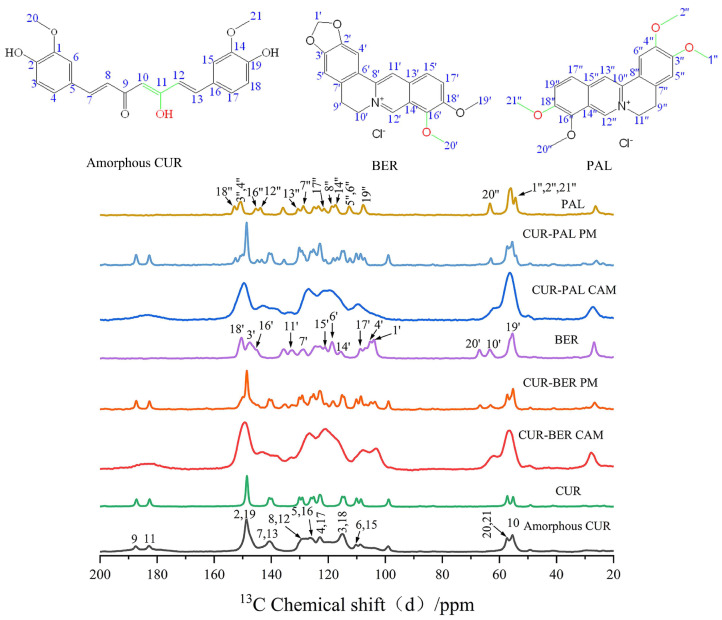
^13^C ssNMR spectra of CUR, BER, PAL, amorphous CUR, CUR-BER CAM, CUR-PAL CAM, and CUR-BER PM and CUR-PAL PM (PM: physical mixture).

To elucidate the intermolecular interactions, particularly hydrogen bonding, between CUR and BER/PAL in the co-amorphous systems, the ^13^C ssNMR spectra of BER, PAL, CUR, amorphous CUR, CUR-BER CAM, CUR-PAL CAM, and the physical mixtures of BER/PAL with CUR (PMs) were recorded (Figure 4). It is well established that curcumin exists in the keto–enol tautomeric form in the solid state [14,33]. For CUR or amorphous CUR, the signals at 183.5 and 187.5 ppm correspond to the carbon atoms C11 (enolic C-OH) and C9 (C=O group), respectively. For BER, the chemical shifts at 145.27 and 150.46 ppm are attributed to C16′ and C18′ attached to methoxy groups, while the peaks at 55.4 and 66.9 ppm correspond to the methyl carbons C19′ and C20′ in the methoxy groups. Similarly, for PAL, the chemical shifts at 145.42, 150.68, and 153.16 ppm correspond to C16″, C3″/C4″, and C18″, all associated with methoxy substituents. The peaks at 54.1 and 63.5 ppm are assigned to the methyl carbons C1″/C2″/C21″and C20″ in the methoxy groups.

Compared with the spectra of individual components (BER, PAL, and CUR), the CUR-BER and CUR-PAL co-amorphous systems exhibited distinct changes, including chemical shift variations, peak broadening, and disappearance of certain resonances. In contrast, the PMs showed no notable changes relative to the pure APIs, suggesting that such interactions only occur in the co-amorphous state.

In the ^13^C ssNMR spectra of CUR-BER CAM and CUR-PAL CAM, marked chemical shifts were observed for the CUR carbon atoms C9 and C11, with the corresponding peaks merging into a broader resonance. This feature indicates an alteration in the conjugation between the keto and enol groups of curcumin, consistent with previously reported CUR-L-arginine and CUR-piperazine co-amorphous system [19,34]. Moreover, significant chemical shift changes and peak broadening were observed for carbons in close proximity to the enolic C-OH (C10, C8, C12, C7, and C13), suggesting that the enol hydroxyl of CUR likely participates in hydrogen bonding with BER or PAL molecules.

For BER in the CUR-BER CAM, prominent chemical shift changes were detected for C16′ and C20′ (methoxy groups), as well as neighboring carbons C14′, C17′, C18′, and C19′. Similarly, in the CUR-PAL CAM, shifts were evident for C1″/C2″/C21″, C18″, and C3″/C4″, implying that at least one of the three methoxy groups in PAL is involved in molecular interactions with CUR.

**Figure 5:**
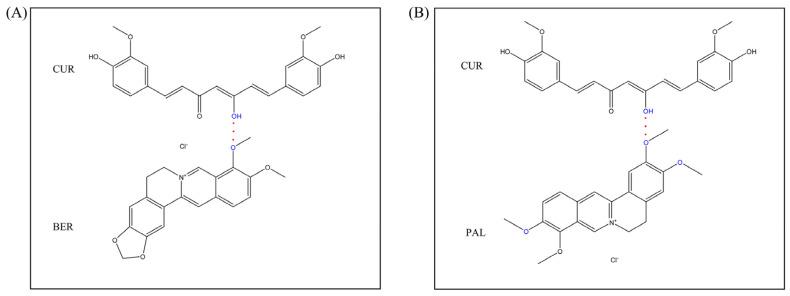
Possible hydrogen-bonding interaction in CUR-BER CAM (**A**) and CUR-PAL CAM (**B**).

Taken together, these results indicate that strong intermolecular hydrogen bonding interactions probably occur between the enolic hydroxyl group of CUR and the oxygen atoms of the methoxy groups in BER or PAL within the co-amorphous systems. The possible hydrogen-bonding interactions are schematically illustrated in Figure 5.

### 3.3. In Vitro Dissolution Study

**Figure 6:**
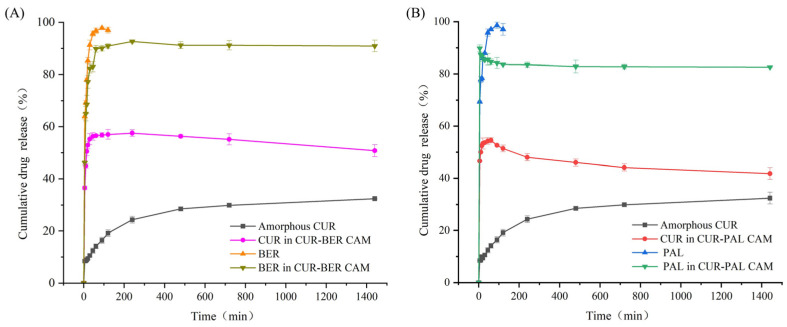
In vitro dissolution profiles of amorphous CUR, CUR in CUR-BER CAM (**A**), and CUR in CUR-PAL CAM (**B**) in water containing 0.5% Tween-80 (*n* = 3).

The dissolution behavior of BER, PAL, amorphous CUR, and the two co-amorphous systems was investigated to evaluate the impact of co-amorphization on drug release performance. Given that non-sink dissolution testing allows the evaluation of the maximum solution concentration attainable by an amorphous material and offers insight into its physical stability during the dissolution process [35], the dissolution studies involving CUR (either as an amorphous API or within co-amorphous systems) were conducted under non-sink conditions. As shown in Figure 6, both BER and PAL displayed rapid dissolution, reaching complete release within 90 min, which can be attributed to their intrinsically high aqueous solubility [36,37]. In contrast, their release from the co-amorphous systems was incomplete throughout the entire dissolution period. This incomplete release may be explained by the strong intermolecular interactions formed between CUR and BER/PAL molecules within the co-amorphous matrices, which can hinder the drug–drug detachment and slow the dissolution process. In addition, partial recrystallization of CUR at the particle surface during dissolution may create a diffusion-limiting layer that impedes the release of the paired molecule.

**Figure 7:**
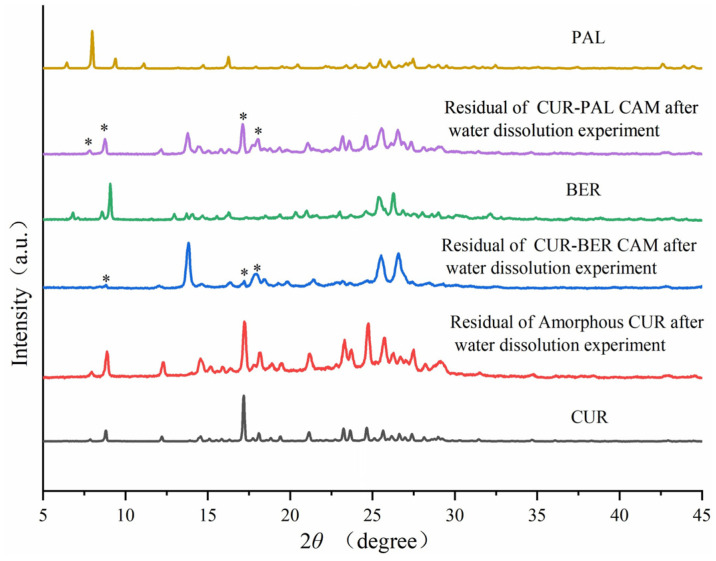
PXRD patterns of residual solid powder after dissolution experiments. The “*” symbols indicate the peak positions corresponding to crystalline CUR.

As shown in Figure 6, amorphous CUR exhibited a comparatively slow dissolution rate, with only 32.4% of the drug dissolved after 24 h. Notably, CUR in both co-amorphous systems showed substantially enhanced dissolution behavior. For the CUR-BER CAM, CUR dissolution reached a maximum of 57.3% at 120 min, whereas for the CUR-PAL CAM, a maximum dissolution of 54.6% was achieved within 60 min. After reaching the peak values, the dissolution of CUR in both co-amorphous systems gradually decreased, showing a characteristic “spring and parachute” profile typical of amorphous formulations [38]. The “spring and parachute” phenomenon describes a rapid rise of drug concentration to a supersaturated level (“spring”), followed by a gradual decline (“parachute”) as the metastable supersaturation relaxes via precipitation or recrystallization. This behavior is common in amorphous systems due to their higher apparent solubility and lower thermodynamic stability [39]. In this study, both CUR-BER and CUR-PAL CAMs exhibited distinct supersaturation peaks and subsequent decreases, consistent with this mechanism. PXRD analysis of the residual solids after dissolution (Figure 7) confirmed that the release process was accompanied by the recrystallization of CUR. This recrystallization explains the declining portion of the “parachute” stage, supporting the supersaturation–precipitation mechanism. These findings clearly indicate that the formation of co-amorphous systems between CUR and BER/PAL significantly enhanced both the dissolution rate and the overall extent of CUR release, suggesting potential bioavailability compared with the pure amorphous CUR.

### 3.4. Equilibrium Solubility Study

The equilibrium solubility of amorphous CUR, CUR-BER CAM, and CUR-PAL CAM was determined in different media containing 0.5% Tween-80, and the results are summarized in Table 1. Amorphous CUR exhibited distinct pH-dependent solubility, increasing from 10.42 ± 1.88 μg/mL at pH 1.2 to 52.65 ± 3.46 μg/mL at pH 6.8. This pH-dependent behavior is consistent with the weakly acidic nature of CUR.

Co-amorphization of CUR with BER or PAL significantly enhanced the solubility of CUR in all tested media, with the CUR-PAL system showing a more pronounced improvement. In deionized water containing 0.5% Tween-80, the solubility of CUR in the CUR-BER CAM and CUR-PAL CAM systems reached 107.2 ± 3.88 μg/mL and 712.82 ± 3.70 μg/mL, respectively, corresponding to approximately 2.3-fold and 15.1-fold improvements relative to amorphous CUR. Similar enhancement trends were observed at other pH values. For example, at pH 1.2, the solubility of CUR-BER CAM and CUR-PAL CAM was 3.5- and 10.3-fold higher than that of amorphous CUR, while at pH 6.8, the respective increases were 2.3- and 11.7-fold.

Despite the substantial solubility improvement, both co-amorphous systems maintained a pH-dependent solubility pattern similar to that of amorphous CUR, suggesting that co-amorphization enhances overall solubility without altering the intrinsic pH sensitivity of CUR.

To further evaluate the intrinsic solubility of the systems, equilibrium solubility was also measured in deionized water and pH-dependent media without 0.5% Tween-80, and the results are summarized in Table S1. As expected, amorphous CUR showed extremely low solubility and was not detectable under Tween-80-free conditions, consistent with its well-known poor aqueous solubility. In contrast, both co-amorphous systems exhibited measurable and markedly improved intrinsic solubility (except for CUR in the CUR-BER CAM system in the pH 1.2 medium). In deionized water, the solubility of CUR in the CUR-BER CAM and CUR-PAL CAM systems reached 44.50 ± 0.03 μg/mL and 68.53 ± 0.17 μg/mL, respectively. Similar enhancement patterns were observed in pH 4.5 and pH 6.8 media.

Although the absolute solubility values obtained in Tween-80–free media were lower than those measured in the presence of 0.5% Tween-80, the relative trend remained unchanged, with CUR-PAL CAM showing the greatest solubility enhancement, followed by CUR-BER CAM and amorphous CUR. These findings indicate that co-amorphization improves not only the apparent solubility in surfactant-containing media but also the intrinsic aqueous solubility of CUR.

### 3.5. Physical Stability Analysis

**Figure 8:**
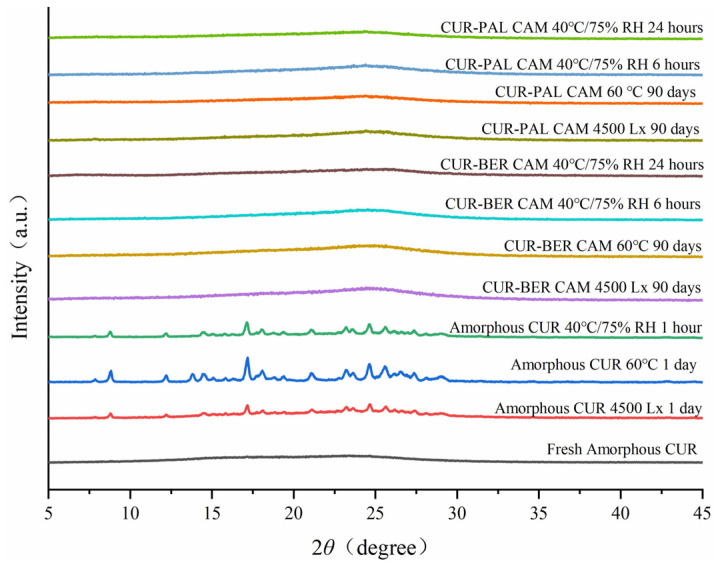
PXRD patterns of amorphous CUR, CUR-BER CAM, and CUR-PAL CAM stored at different stress conditions.

The physical stability of amorphous CUR, CUR-BER CAM and CUR-PAL CAM was evaluated in accordance with the Chinese Pharmacopoeia (2020 Edition) under three stress conditions: 60 °C (monitored for 90 days), light exposure at an intensity of 4500 Lx (monitored for 90 days), and 40 °C/75% relative humidity (RH, monitored for 24 h, due to the known instability of amorphous CUR under moisture) [30]. Structural evolution during storage was monitored by PXRD, and the corresponding diffraction patterns are presented in Figure 8. Under both 60 °C and 4500 Lx conditions, amorphous CUR rapidly recrystallized, showing almost complete conversion to the crystalline form after only one day. In contrast, the PXRD patterns of the two co-amorphous systems retained the characteristic broad and diffuse halos without distinct reflections throughout the 90-day stability study under the same conditions, suggesting the absence or a markedly slower rate of crystallization. At 40 °C/75% RH, amorphous CUR recrystallized within one hour, whereas both co-amorphous systems exhibited no detectable crystallization within 24 h. These results clearly demonstrate that humidity exerts a pronounced influence on the physical stability of amorphous forms. Furthermore, strong intermolecular interactions between CUR and BER, as well as between CUR and PAL, increase the energy barriers for molecular rearrangement, thereby delaying the recrystallization of the co-amorphous systems.

### 3.6. Antioxidant Assay

The antioxidant activities of CUR, BER, PAL, and the two co-amorphous systems were assessed using DPPH free radical-scavenging and ABTS^+^ radical-scavenging assays. The DPPH radical employed in this study is a stable, neutral radical at ambient temperature. In ethanol solution, DPPH exhibits an intense purple color that gradually fades upon reduction by antioxidants [40]. The ABTS radical, in contrast, is a cationic substrate that forms blue-green ABTS^+^ radicals characterized by a distinct absorption maximum at 734 nm. The presence of antioxidants inhibits the generation of ABTS^+^, resulting in a decrease in absorbance [41].

**Figure 9:**
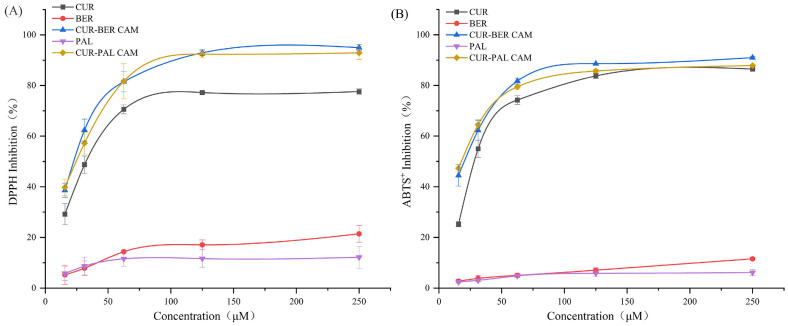
Radical scavenging activity of CUR-BER CAM and CUR-PAL CAM against DPPH (**A**) and ABTS^+^ (**B**) at various drug concentrations (*n* = 3).

As illustrated in Figure 9A, both CUR-BER CAM and CUR-PAL CAM exhibited stronger DPPH radical-scavenging activity than their individual components across the tested concentration range (15.6–250 μM). Moreover, both CAMs displayed a pronounced synergistic effect at lower concentrations. A similar trend was observed in the ABTS^+^ radical-scavenging assay (Figure 9B), where both co-amorphous systems showed enhanced antioxidant performance compared to the pure APIs. Collectively, these findings indicate that the two CAMs possess superior antioxidant capacity, with evident synergistic behavior at low concentrations. This synergism highlights the promising potential of the CAMs as effective antioxidant agents for mitigating free radical-induced oxidative stress in biological systems.

### 3.7. Anticancer Activity Analysis

**Figure 10:**
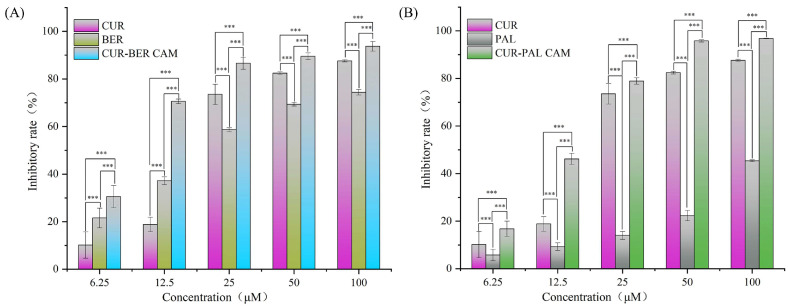
Inhibitory rates of CUR, BER, and CUR-BER CAM (**A**), and CUR, PAL, and CUR-PAL CAM (**B**) on HT-29 cells. Differences were considered statistically significant at *** *p* < 0.001.

The anticancer activities of all samples were evaluated using a cell proliferation assay in the human colorectal adenocarcinoma cell line HT-29. As shown in Figure 10, the inhibitory effects of CUR, BER, PAL, CUR-BER CAM, and CUR-PAL CAM on HT-29 cells increased progressively with rising drug concentrations. In addition, both CUR-BER CAM and CUR-PAL CAM exhibited higher inhibitory effects than their respective single components across the concentration range of 6.25–100 μM, demonstrating the enhanced cytotoxic performance. Notably, at a concentration of 12.5 μM, the inhibition rates of CUR-BER CAM and CUR-PAL CAM exceeded the sum of those of the individual components, indicating enhanced combined activities between the two compounds at this drug concentration. Furthermore, the IC_50_ value of CUR against HT-29 cells was 18.2 μM, whereas those of BER and PAL were 20.0 μM and 142.4 μM, respectively. Remarkably, the IC_50_ values of CUR-BER and CUR-PAL CAMs decreased substantially to 7.9 μM and 14.9 μM, respectively, confirming their enhanced cytotoxic efficacy relative to the individual components. These findings highlight the synergistic potential of co-amorphization in improving the anticancer activity of CUR in combination with isoquinoline alkaloids.

## 4. Conclusions

In this study, co-amorphous systems of curcumin with berberine hydrochloride (CUR-BER CAM) and palmatine hydrochloride (CUR-PAL CAM) were successfully developed. Solid-state characterizations, including PXRD, DSC, SEM, and ssNMR, confirmed the formation of co-amorphous phases, likely stabilized through strong intermolecular hydrogen bonding between the enolic hydroxyl group of CUR and the oxygen atoms of the methoxy groups in BER or PAL. Both CAMs exhibited markedly improved physical stability over amorphous CUR, as evidenced by elevated glass transition temperatures and effective suppression of recrystallization under thermal and light stress conditions (for 90 days), together with humidity stress conditions (for 24 h). Moreover, the CUR-BER and CUR-PAL CAMs showed significantly enhanced solubility and faster dissolution rates compared with amorphous CUR. In addition, both systems displayed superior antioxidant activity and improved anticancer effects against HT-29 cells. Overall, this study demonstrates that co-amorphization of curcumin with isoquinoline alkaloids offers a promising strategy to simultaneously enhance the physicochemical properties of curcumin and improve multifunctional physiological activities.

## Figures and Tables

**Table 1:** Equilibrium solubility data of amorphous CUR, CUR in CUR-BER CAM, and CUR-PAL CAM (*n* = 3).

Dissolution Media ^1^	Amorphous CUR (μg/mL)	CUR in CUR-BER CAM (μg/mL)	CUR in CUR-PAL CAM (μg/mL)
deionized water	47.09 ± 4.89	107.2 ± 3.88	712.82 ± 3.70
pH 1.2	10.42 ± 1.88	36.26 ± 1.19	107.47 ± 2.99
pH 4.5	32.91 ± 2.56	72.05 ± 0.29	426.09 ± 2.73
pH 6.8	52.65 ± 3.46	120.6 ± 8.66	615.88 ± 13.30

^1^ All dissolution media contained 0.5% Tween-80.

## Data Availability

The original contributions presented in this study are included in the article/Supplementary Materials. Further inquiries can be directed to the corresponding author.

## Supplementary Materials

The following supporting information can be downloaded at: https://www.mdpi.com/article/10.3390/pharmaceutics18010009/s1, Figure S1: PXRD patterns of the samples with CUR-to-BER/PAL molar ratios at 2:1 and 1:2.; Table S1: Equilibrium solubility data of amorphous CUR, CUR in CUR-BER CAM, and CUR-PAL CAM (*n* = 3) measured in deionized water and pH-dependent media without 0.5% Tween-80.

## Abbreviations

The following abbreviations are used in this manuscript:

APIs	active pharmaceutical ingredients
BER	berberine hydrochloride
CAM	co-amorphous
CP	cross-polarization
CUR	curcumin
DSC	differential scanning calorimetry
HPLC	high-performance liquid chromatography
IR	inhibition rate
MAS	magic angle spinning
OD	optical density
PAL	palmatine hydrochloride
PBS	phosphate-buffered saline
PM	physical mixture
PXRD	powder X-ray diffraction
SD	standard deviation
ssNMR	solid-state nuclear magnetic resonance
