# Supplementary material for: Two Drug–Drug Co-Amorphous Systems of Curcumin and Berberine Hydrochloride/Palmatine Hydrochloride with Improved Physicochemical Properties and Multifunctional Activities
Source: Pharmaceutics. 2025 Dec 20;18(1):9. doi: 10.3390/pharmaceutics18010009 (PMC12845240; doi:10.3390/pharmaceutics18010009)
Supplement: Supplementary file 1 [file pharmaceutics-18-00009-s001.zip › pharmaceutics-3991690-supplementary.pdf]

pharmaceutics

MDPI

Supporting Information

Two Drug–drug Co-amorphous Systems of Curcumin and Berberine Hydrochloride /Palmatine Hydrochloride with Improved Physicochemical Properties and Multifunctional Activities

Yanjie Zhang *, Quanhu Guo, Ling Liang, Mei Zhang, Rongjian Sa and Benyong Lou *

College of Materials and Chemical Engineering, Minjiang University, Fuzhou, Fujian, 350108, China
* Correspondence: yanjiezhang@mju.edu.cn; lby@mju.edu.cn

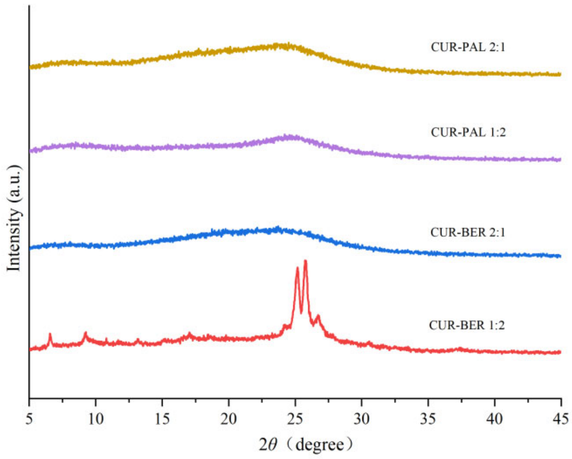

Figure S1. PXRD patterns of the samples with CUR-to-BER/PAL molar ratios at 2:1 and 1:2.

Table S1. Equilibrium solubility data of amorphous CUR, CUR in CUR-BER CAM, and CUR-PAL CAM (n = 3) measured in deionized water and pH-dependent media without 0.5% Tween-80.

Dissolution media	Amorphous CUR (μg/mL)	CUR in CUR-BER CAM (μg/mL)	CUR in CUR-PAL CAM (μg/mL)
deionized water	ND	44.50±0.03	68.53±0.17
pH 1.2	ND	ND	50.54±0.01
pH 4.5	ND	37.51±0.02	84.84±0.09
pH 6.8	ND	38.91±0.03	82.04±0.03
